# Cholecalciferol Supplementation Impacts Behavior and Hippocampal Neuroglial Reorganization in Vitamin D-Deficient Rats

**DOI:** 10.3390/nu16142326

**Published:** 2024-07-19

**Authors:** Zsolt Gáll, Ágnes Csüdör, István-Gábor Sável, Krisztina Kelemen, Melinda Kolcsár

**Affiliations:** 1 Department of Pharmacology and Clinical Pharmacy, George Emil Palade University of Medicine, Pharmacy, Science, and Technology of Targu Mures, 540142 Târgu Mureș, Romania; melinda.kolcsar@umfst.ro; 2 Faculty of Medicine, George Emil Palade University of Medicine, Pharmacy, Science, and Technology of Targu Mures, 540142 Târgu Mureș, Romania; csudoragnes@gmail.com; 3 Faculty of Pharmacy, George Emil Palade University of Medicine, Pharmacy, Science, and Technology of Targu Mures, 540142 Târgu Mureș, Romania; savelistvan@gmail.com; 4 Department of Physiology, George Emil Palade University of Medicine, Pharmacy, Science, and Technology of Targu Mures, 540142 Târgu Mureș, Romania; krisztina.kelemen@umfst.ro

**Keywords:** vitamin D deficiency, cholecalciferol, paricalcitol, cognitive dysfunction

## Abstract

Vitamin D deficiency (VDD) is widespread around the world and has been extensively documented to affect various health conditions, including the cognitive functioning of the brain. Serum 25-hydroxylated forms of vitamin D are traditionally used to determine vitamin D status. However, there is now evidence that cholecalciferol activation can occur and be controlled by locally expressed enzymes in the brain. This study aimed to investigate the effects of cholecalciferol supplementation on cognitive function in rats who underwent transient VDD in adulthood. Thirty-six adult Wistar rats were administered paricalcitol (seven doses of 32 ng injected every other day) along with a “vitamin D-free” diet to induce VDD, which was confirmed using a LC–MS/MS serum analysis of the cholecalciferol and 25-hydroxyvitamin D3 levels. Treatment was performed by including 1000 IU/kg and 10,000 IU/kg cholecalciferol in the diet. Cognitive performance was evaluated using the novel object recognition (NOR), Morris water maze (MWM), and radial arm maze (RAM) tests. An immunohistochemical analysis of the brain regions involved in learning and memory was performed by quantifying the neurons, astrocytes, and microglia labelled with anti-neuronal nuclei (NeuN), glial fibrillary acidic protein (GFAP), and ionized calcium-binding adaptor molecule 1 (Iba-1) antibodies, respectively. The vitamin D deficient group showed the lowest performance in both the MWM and RAM tests. In contrast, the cholecalciferol-treated groups exhibited a faster learning curve. However, no difference was detected between the groups in the NOR test. On the other hand, differences in the cellular organization of the hippocampus and amygdala were observed between the groups. Cholecalciferol supplementation decreased the density of the Iba-1- and GFAP-labeled cells in the hilus and cornu Ammonis 3 (CA3) regions of the hippocampus and in the amygdala. These results support vitamin D’s substantial role in learning and memory. They also highlight that subtle changes of cognitive function induced by transient VDD could be reversed by cholecalciferol supplementation. Further studies are needed to better understand VDD and cholecalciferol’s effects on the brain structure and function.

## 1. Introduction

Vitamin D deficiency (VDD) is widespread around the world, and in many parts of the world, it has even been considered as a prevalent public health issue [1]. Vitamin D can either be ingested in the diet as cholecalciferol (vitamin D3) and ergocalciferol (vitamin D2) or synthesized in the skin when exposed to sunlight. The current evaluation of vitamin D status relies on the measurement of the major circulating form, 25-hydroxyvitamin D3 (25-OH-D3), and its conversion to the active hormonal form, 1α,25-dihydroxyvitamin D3 (1,25-(OH)2-D3); the latter metabolite is usually present at very low concentrations and may only be present in specific target tissues, such as the kidney, immune cells, and certain types of carcinomas [2].

In the past two decades, several experimental and clinical studies have confirmed the importance of vitamin D in the central nervous system (CNS), and 1,25-(OH)2-D3 is now considered as a neurosteroid with multiple effects [3]. The link between serum 25-OH-D3 levels and cognitive performance has been established in both rodents and humans, with decreased levels observed in patients with dementia and age-related cognitive decline [4,5,6,7].

It is known that both vitamin D3 and its metabolites (25-OH-D3 and 1,25-(OH)2-D3) cross the blood–brain barrier and their receptor, the vitamin D receptor (VDR), is abundantly expressed across different brain regions [8,9,10]. Furthermore, it was found that neurons express the enzymes responsible for the second hydroxylation of 25-OH-D3 (CYP27B1, CYP24A1), suggesting that neuronal signaling (an autocrine/paracrine system within the nervous system) could govern vitamin D signaling independently without the systemic context significantly influencing it [11,12]. Moreover, cholecalciferol can be converted to 25-OH-D3 in nearly all cerebral cell types, and CYP27A1 has been found to be expressed in astrocytes, microglia, and oligodendrocytes. Besides, a membrane receptor (protein disulfide isomerase family member 3—PDIA3) has recently been proposed as a mediator of some of the non-genomic effects of vitamin D in the brain, showing an abundant expression in all types of cells (neurons, glia cells, endothelial cells). Landel et al. (2018) demonstrated that PDIA3 receptors are involved in diosgenin-induced cognitive enhancement in mice, and that their activation reduced amyloid plaque accumulation in an Alzheimer’s animal model [12]. The nongenomic effects of vitamin D via PDIA3 receptors and membrane-bound VDR include the initiation of an influx of Ca^2+^, the rapid intracellular release of Ca^2+^ stores, the modulation of adenylate cyclase and phospholipase C, and protein phosphorylation [13,14].

Many studies focused on the effects of vitamin D metabolites delivered directly to the brain, and great progress has been made regarding the neurodevelopmental effects of vitamin D, particularly in the hippocampus, which plays a crucial role in memory and learning [15]. However, there is limited evidence that increasing systemic vitamin D3 exposure results in cognitive enhancement in adult rats. The pharmacokinetics of vitamin D metabolites in the brain and the impact of systemic vitamin D insufficiency and/or deficiency on the central nervous system have not been adequately explored yet. A recent study in rats confirmed that vitamin D3 supplementation increases the concentration of 25-OH-D3 in the brain homogenate, resulting in a brain-to-serum ratio of 0.25 independent of the serum levels [16]. On the other hand, the behavior of rats and mice subjected to dietary developmental VDD showed alterations such as disrupted attentional processing and learning deficits [17,18,19,20]. However, the effects of transient VDD and cholecalciferol supplementation on adult rats remain controversial. The vitamin D-free diet induces a slow decrease in serum 25-OH-D3 in middle-aged adult rodents, but cognitive deficits were not detected even after an extended period of vitamin D deprivation [17,21,22]. Conversely, administering different doses of cholecalciferol to aging rats showed protective effects against mild cognitive impairment [23] and improved the long-term memory of mice [24].

This study aimed to assess the influence of cholecalciferol administration on the behavioral characteristics of vitamin D-deficient adult rats and investigated the cellular modifications of the brain regions involved in cognition and learning. Adult rats were exposed to transient VDD as described by Stavenuiter et al., followed by a specific diet [25]. Using this approach, the seasonal fluctuations in vitamin D exposure might be modelled more precisely. The main objective of the current study was to examine whether vitamin D supplementation could improve VDD rats’ altered cognitive performance.

## 2. Materials and Methods

### 2.1. Animals

A total of 36 male 6-month-old Wistar rats weighing 370–420 g at the beginning of the experiment were used. All animals were housed in groups of 3–4 per cage (1500 U Eurostandard Type IV S cages; 480 × 375 × 210 mm; Tecniplast SpA., Buguggiate, Italy) and maintained under standard laboratory conditions of a 12-h dark–light cycle and controlled temperature (20 ± 2 °C) and humidity (40–60%), with unlimited access to food and water. All animals underwent a vitamin D deficiency induction, using a method previously described [25]. Briefly, the rats were fed a vitamin D-free diet for 14 days. Meanwhile, they received repeated intraperitoneal injections of paricalcitol (on days 1, 3, 5, 7, 9, 11, and 13) at a dose level of 32 ng dissolved in 0.9% saline, in order to induce CYP24A1 expression and accelerate the catabolism of endogenous 25-OH-D3 and 1,25-(OH)2-D3 stores. The dose of paricalcitol, the frequency of administration, and the diet composition were the same as described by Stavenuiter et al. [25], followed by the validation of VDD after 4 weeks.

**Figure 1:**
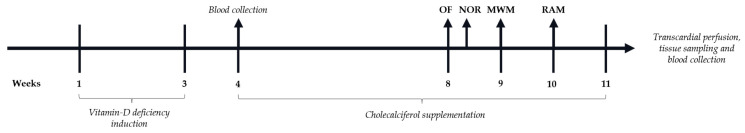
Schematic illustration of the experimental design and timeline of the study. Abbreviations: OF, open field test; NOR, novel object recognition test; MWM, Morris water maze test; RAM, radial arm maze test.

Afterward, the animals were randomly divided into three groups receiving various diets. The vitamin D deficient group (VDD, n = 12) continued to receive a vitamin D-free diet; the second group (LVD, n = 12) was supplemented with low vitamin D dose (cholecalciferol 1000 IU/kg included in the standard diet); the third group (HVD, n = 12) received a higher dose of cholecalciferol (10,000 IU/kg in the diet). The cholecalciferol dose selection was based on previous studies in rats, where significant effects on cognitive performance were described [22,23]. Specific dietary formulas were given for a 7-week period, in which the cognitive abilities of the animals were assessed by subjecting them to behavioral tests. The comprehensive timeline for this experiment can be seen in Figure 1.

All the procedures with animals were performed according to the European Community Guidelines for the Use of Experimental Animals (Directive 2010/63/EU) and the applicable national and local guidelines. The rats were procured from the Biobase of UMFST George Emil Palade of Targu Mures, Romania. The study protocol was previously approved by the local ethics committee (decision no. 391/04.10.2019) and the national authorities (project no. 48/16.02.2021). The order of the treatments and measurements or the animal/cage location was random to minimize potential confounders. No exclusion criteria were predetermined, and no animals were excluded or died during the experiment.

### 2.2. Drugs and Reagents

#### 2.2.1. Drugs and Reagents

Paricalcitol (TRC-P195300, TRC, Burlington, ON, Canada) was purchased from LGC Standards (Manchester, NH, USA) and dissolved in a sterile saline solution (Braun, Jaén, Spain). The vitamin D-free diet and the specific diet formulas containing 1000 IU/kg and 10,000 IU/kg vitamin D3 were prepared by the Cantacuzino Institute (Bucharest, Romania). Vacutainer serum tubes (BD #367812, Becton Dickinson, Basel, Switzerland) were used for the serum sampling. The solvents used for sample preparation were LC-grade. Methanol (catalog #60-009-47, ChromaSolv^®^, Sigma-Aldrich, St. Louis, MO, USA), n-hexane (BDH24575.100E, Hipersolv Chromanorm^®^, Sigma-Aldrich, St. Louis, MO, USA), methyl tert-butyl ether (catalog #60-016-45, ChromaSolv^®^, Sigma-Aldrich, St. Louis, MO, USA), and acetonitrile (catalog # 60-046-514, ChromaSolv^®^, Sigma-Aldrich, St. Louis, MO, USA) were used for the analyte extraction. For the mobile phase, formic acid (Scharlau, Sentmenat, Spain) and ultrapure water (Millipore Direct-Q water purification system, Millipore, Bedford, MA, USA) were used.

#### 2.2.2. Liquid Chromatography–Mass Spectrometry (LC–MS/MS)

The LC–MS/MS method was used for the quantification of cholecalciferol and 25-OH-D3 in serum and brain homogenate samples. The method was developed and validated for different types of biological matrices as published previously [26]. Briefly, serum samples were spiked with internal standard solutions and the proteins were precipitated with acetonitrile (1:1 *v*/*v*). The analytes of interest were concentrated using liquid–liquid extraction with n-hexane and methyl tert-butyl ether, followed by evaporation (Thermo Scientific Savant SpeedVac Concentrator, SPD121P, ThermoFisher Scientific, Marietta, OH, USA). The dry residue was dissolved in acetonitrile and the analytes underwent a Diels–Alder derivatization with 4-phenyl-1,2,4-triazoline-3,5-dione (PTAD, LiChropur for LC-MS, Sigma-Aldrich, Darmstadt, Germany) in acetonitrile. The final mixture was allowed to complete the derivatization reaction while protected from light and left to stand at room temperature overnight.

The LC–ESI–MS/MS method was developed to measure 25-OH-D3 and cholecalciferol obtaining a limit of detection of 0.1 ng/mL. The internal standard method was used for the quantification of each analyte using d3-vitamin D3 (6,19,19-d3) (product #740284, Cerilliant, Round Rock, TX, USA) and d6-25(OH)D3 (26,26,26,27,27,27-d6) (product #H-074, Cerilliant, Round Rock, TX, USA) as standard references.

### 2.3. Behavioral Tests

Animal models attempt to reproduce the features of human psychiatric disorders in laboratory animals, correlating the physiological and behavioral changes associated with specific emotional states (face validity), the etiology of diseases (construct validity), and responses to pharmacological treatments (predictive validity) [27]. Behavioral tests can measure the cognitive functions, learning, and memory of laboratory animals using their innate characteristics. 

Throughout the 7-week-long treatment period the animals underwent four neurobehavioral tests chosen to evaluate anxiety-like behavior and measure hippocampal-dependent and -independent learning and memory. Initially, the open field (OF) test was employed to assess the anxiety level of all the groups, since anxiety could potentially affect the performance in learning and memory tests. This was followed by the novel object recognition (NOR), Morris water maze (MWM), and radial arm maze (RAM) tests, probably the most employed animal models for learning and memory [28]. In order to allow the animals to recover after each exposure to stress, three tests assessing cognitive function were performed over 3 weeks. At the beginning of each trial, the order of the trials was randomized. No blinding methods were employed for the behavioral tests.

#### 2.3.1. Open Field (OF) Test

The OF test is a fast and relatively easy test that can provide a variety of information regarding the general locomotor ability and anxiety-related emotional behaviors of rodents. The rats were individually placed in the center of a 60 × 60 × 50 cm open field arena and left free to explore the area for 5 min. The activity was recorded using a CMOS video camera (30 fps) located above and analyzed using EthoVision XT (version 11.5, Noldus IT, Wageningen, The Netherlands). Locomotion was determined by measuring the total distance traveled in the open field and anxiety-related behavior was evaluated using the time spent in the center of the open field. 

#### 2.3.2. Novel Object Recognition (NOR)

The NOR test is based on the natural propensity of rats to explore novel objects. It is a simple behavioral assay of memory that relies primarily on a rodent’s innate exploratory behavior in the absence of externally applied rules or reinforcement [28].

All the animals were given a 5-min-long habituation in the same arena that was used in the OF test with no object. Twenty-four hours later the animals were allowed to explore two identical objects, consistently placed at the same location inside the box for a total of 5 min (training session). The familiar objects were grey, concrete blocks with a dimension of 20 × 10 × 6 cm. After this phase, the animals were returned to their home cages. Memory retention was assessed during the test session, which was carried out 4 h after the training session. One familiar and one novel object were presented to the animals. The novel object was a light green plastic cube toy with similar dimensions (18 × 10 × 6 cm). Both objects were fixed to the arena floor with glue to assure that the animals could not move them. We chose objects that were different enough to be easily distinguished by the rats but were similar in shape and size and had a similar degree of complexity in order to minimize any potential object preference that may bias the results [29].

**Figure 2:**
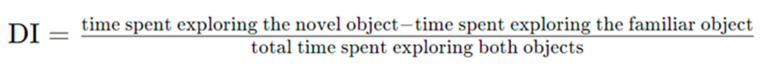
Discrimination index. The differential exploration of two items was quantified by calculating a discrimination index (DI), in which the difference in the amount of time spent with one object vs. the other is expressed as a proportion of the total exploration time. A positive DI indicates a preference for exploring the novel object, suggesting intact memory retention and recognition of the previously encountered familiar object. Conversely, a negative or zero DI may suggest impaired memory function or lack of recognition.

Following each test, the arena was thoroughly cleaned with 70% ethanol solution. The exploration time was defined as sniffing or touching the object with the nose and was quantified afterward. The discrimination index was calculated for the retention trials using the formula presented in Figure 2.

#### 2.3.3. Morris Water Maze (MWM)

The Morris Water Maze (MWM) test is a classic behavioral paradigm used to assess spatial learning and memory in rodents, particularly in mice and rats [30].

In our study, we utilized a circular pool (90 cm in diameter, 60 cm deep) filled with opaque water, in which a hidden platform (9 cm in diameter) was submerged just beneath the surface. The pool was divided into four quadrants, and distinct visual cues were placed around the room to aid spatial navigation [31]. 

During the learning phase, which lasted for 4 days, the animals underwent four trials per day, with each trial lasting for 120 s. The intertrial period was 1 min. The animals were tasked with locating the hidden platform within the allotted time, using spatial cues to guide their search. Entry from four different starting positions was varied in a random order. If an animal failed to find the platform within the given timeframe, it was gently guided to the platform to reinforce the spatial association. On the fifth day, the testing phase commenced. The hidden platform was removed, and the animals were released from the quadrant stated opposite to the target quadrant and given one 30-s retention swimming trial. 

Exploration behavior was recorded using video tracking software, enabling a precise analysis of specific parameters, including the latency to reach the platform location, the time spent in the target quadrant, and the total distance traveled. 

#### 2.3.4. Radial Arm Maze (RAM)

The radial arm maze is a spatial navigation memory task involving primarily the hippocampus and prefrontal cortex. This test has been most extensively used to investigate specific aspects of spatial working and reference memory [32]. 

The apparatus consisted of a round center platform with eight arms radiating outward in a symmetrical arrangement. Six out of the eight arms were baited with food rewards at the distal end, motivating the animals to explore the maze. The sequence of baiting (arms 2, 3, 4, 5, 7, and 8) remained constant throughout the experiment.

The training occurred over four consecutive days, with one trial per day, and this was followed by a 3-day-long testing period. Each animal was placed individually into the central platform and was allowed to move freely in the maze and retrieve the food rewards. The trial ended when all the available cereal rewards had been collected or after a predetermined time limit (10 min). Researchers observed the experiment in the next room without contact with or influence on the animal. Following each trial, the apparatus was cleaned with 70% ethanol solution to avoid any potential olfactory bias.

A reference memory error was scored when the rat entered an arm that was not baited, since the animals would need to recall from the previous days which arms were not baited to avoid error. A working memory error was scored as reentry into an arm from which the bait had already been eaten, since the animals would have to recall within a given trial which arms they had already visited. 

### 2.4. Serum and Brain Sampling

#### 2.4.1. Vitamin D Status and Biochemical Parameters

The rats were anesthetized with 3% isoflurane and 300 μL of peripheral blood was collected from the tail vein in serum tubes for the determination of the basal vitamin D status using LC–MS/MS. At the end of the experiment, blood samples were collected by cardiac puncture into serum tubes. Whole blood was centrifuged at 2000× *g* for 10 min at 4 °C to obtain serum. Serum samples were used for the determination of the following parameters (units of measure): aspartate aminotransferase (AST, U/L), alanine aminotransferase (ALT U/L), alkaline phosphatase (ALP, U/L), gamma-glutamyl transferase (GGT, U/L), total bilirubin (mg/dL), albumin (g/dL), globulin (g/dL), total protein (g/dL), creatinine (mg/dL), blood urea nitrogen (BUN, mg/dL), total cholesterol (mg/dL), glucose (mg/dL), calcium (mg/dL), phosphorus (mg/dL), potassium (K, mmol/L), and sodium (Na, mmol/L). Biochemical analyses were performed using a fully automated veterinary chemistry analyzer (Element RC, Heska Corporation, Loveland, CO, USA).

#### 2.4.2. Preparation of Tissues

The rats were anesthetized with a mixture of ketamine–xylazine (100 mg/kgbw and 10 mg/kgbw) administered intraperitoneally. Transcardial perfusion was performed with ice-cold 0.9% saline solution, followed immediately by a fixative containing 4% paraformaldehyde (catalog #158127-500G, Sigma-Aldrich, St. Louis, MO, USA) in 0.1 M phosphate buffer (PB, pH 7.4, catalog #P3619, Sigma-Aldrich, St. Louis, MO, USA) for 20 min. The brains were removed from the skull and postfixed in the same fixative solution for 24 h at 4 °C, then immersed in 0.1 M PB until sectioning. Each tissue block was sectioned serially at a thickness of 60 μm using a vibratome (VT 1000S, Leica, Nussloch, Germany). In this study, coronal brain slices located at approximately 6.2 and 5.4 mm rostral from the interaural line were chosen. 

#### 2.4.3. Immunohistochemical Staining

Immunofluorescent staining was performed on free-floating sections to visualize the neuron–astrocyte–microglia triad using the following established markers: neuron-specific nuclear protein (NeuN) for the neurons, glial fibrillary acidic protein (GFAP) for the astrocytes, and ionized calcium-binding adaptor molecule 1 (Iba-1) for the microglia. 

The brain sections were rinsed in 0.1 M PB for 10 min at room temperature, then washed twice for 20 min in a Tris-buffered saline (TBS, catalog#T6664, Sigma-Aldrich, St. Louis, MO, USA). In order to block nonspecific protein binding and enhance antibody penetration, the slices were incubated for 45 min in TBS containing 10% normal horse serum (NHS; catalog #S-2000-20, Vector Laboratories, Burlingame, CA, USA) and 0.3% Triton-X (catalog #X100, Sigma-Aldrich, St. Louis, MO, USA). This was followed by an overnight incubation in 0.1% TBS-T with primary antibodies, including the guinea pig anti-NeuN antibody (1:500, guinea pig raised-polyclonal, product no: 266004, Synaptics Systems GmbH, Goettingen, Germany), the mouse anti-GFAP antibody (1:500, mouse raised-monoclonal; product no: 173211, Synaptics Systems GmbH, Goettingen, Germany), and the rabbit anti-Iba-1 antibody (1:500, rabbit raised-polyclonal; HS234013, Synaptics Systems GmbH, Goettin-gen, Germany). 

After repeated washes in TBS, the sections were incubated with the following corresponding secondary antibodies for 4 h at room temperature: the Alexa488-conjugated anti-guinea pig antibody (1:400, catalog #106-545-003, Jackson ImmunoResearch Laboratories, West Grove, PA, USA), the Alexa594-conjugated anti-mouse antibody (1:400, catalog #715-585-150, Jackson ImmunoResearch Laboratories, West Grove, PA, USA), and the Cy3-conjugated anti-rabbit (1:400, catalog #111-165-003, Jackson ImmunoResearch Laboratories, West Grove, PA, USA) antibody. In addition to the secondary antibodies, 4′,6-diamidino-2-phenylindole (DAPI) was applied in a 1:2000 dilution to label the total cell nuclei. The sections were then washed three times in TBS for 20 min and twice in PB for 10 min. Afterwards, we mounted each histological section on glass slides and coverflipped them using a Vectashield mounting medium (Vectashield, Vector Laboratories, Burlingame, CA, USA).

#### 2.4.4. Evaluation of Immunostaining and Analysis

To provide quantitative measurements, whole-slide image acquisition was performed using immunohistochemistry with a Pannoramic Scan digital slide scanner (3DHistech, Budapest, Hungary). The average scanning parameters were 20× magnification, using a Plan-Apochromat objective with a micrometer/pixel ratio of 0.325000. The images were processed using the Slideviewer software (version 2.6.0.166179, 3DHistech, Budapest, Hungary) and evaluated by two researchers. To reduce the interobserver variability first, the two researchers independently examined and scored the scanned images. In the case of disagreement, the questionable cases were discussed to reach a final conclusion and scoring. 

**Figure 3:**
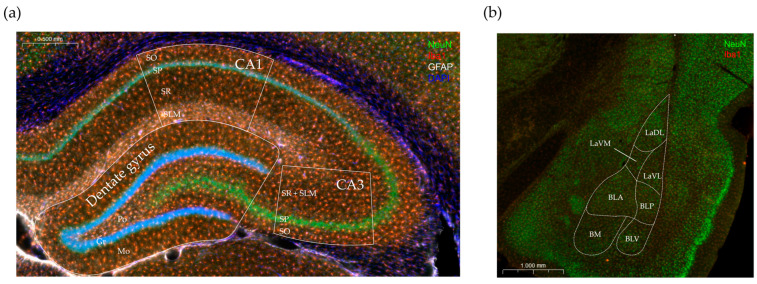
Representative photomicrographs of an immunostained coronal brain section, highlighting the regions of interest, where the cell quantification was performed. (**a**) Hippocampus: This region consists of the cornu Ammonis (CA) and the dentate gyrus. Cells were quantified in predefined areas within the CA1 and CA3 regions, specifically in each of the following layers: *stratum oriens* (SO), *stratum pyramidale* (SP), *stratum radiatum* (SR), and *stratum lacunosum moleculare* (SLM). The dentate gyrus is composed of three layers (Mo—molecular layer; Gr—granular layer; Po—polymorphic layer). Cells were quantified exclusively in the polymorphic layer, also known as the hilus. (**b**) Amygdala: The schematic illustration indicates the localization of the nuclei (LaDL—lateral amygdaloid nucleus, dorsolateral portion; LaVM—lateral amygdaloid nucleus, ventromedial portion; LaVL—lateral amygdaloid nucleus, ventrolateral portion; BLA—basolateral amygdaloid nucleus, anterior portion; BLP—basolateral amygdaloid nucleus, posterior portion; BM—basomedial amygdaloid nucleus; BLV—basolateral amygdaloid nucleus, ventral portion).

Two coronal slices per animal were used for the histological assessment. Manual cell counting was performed in the polymorphic layer of the dentate gyrus and all the layers of the cornu Ammonis 1 (CA1) and cornu Ammonis 3 (CA3) regions of the hippocampus, including the stratum oriens, the stratum pyramidale, the stratum radiatum, and the stratum lacunosum moleculare (Figure 3a). We also analyzed the amygdaloid area containing the basolateral amygdaloid nucleus, the anterior and posterior parts (BLA, BLP), and the lateral amygdaloid nucleus ventrolateral part (LaVL) (Figure 3b). The regions of interest were identified using the Paxinos and Watson rat brain atlas and analyzed by two blinded experimenters. We counted the various cell types consistently within the same area on each slice and calculated the cell density (cells/mm^2^). 

### 2.5. Statistical Analysis

A sample size calculation was performed a priori using G*Power (version 3.1.9.7.; University of Duesseldorf, Duesseldorf, Germany) with α = 0.05 and 1 − β = 0.95. Based on Latimer et al. [23], for the main parameter of the MWM test, an effect size of 0.713 and a standard deviation of 5 were calculated. This resulted in a minimum total sample size of 36 animals. Data were analyzed for a normal distribution using the Kolmogorov–Smirnov test (GraphPad Prism 8, GraphPad Software, San Diego, CA, USA). Data with a Gaussian distribution were presented as mean ± SEM and analyzed using an ordinary one-way ANOVA test. Nonparametric distributed data were expressed as median (interquartile range) and analyzed using the Kruskal–Wallis test followed by Dunn’s post hoc test to detect the significant differences between the groups. For the repeated parameters (e.g., MWM and RAM tests), the analysis was performed using a two-way ANOVA followed by Tukey’s multiple comparisons test. *p* < 0.05 was considered statistically significant.

## 3. Results

### 3.1. Pharmacokinetic and Biochemical Determinations

#### 3.1.1. Vitamin D Status

**Figure 4:**
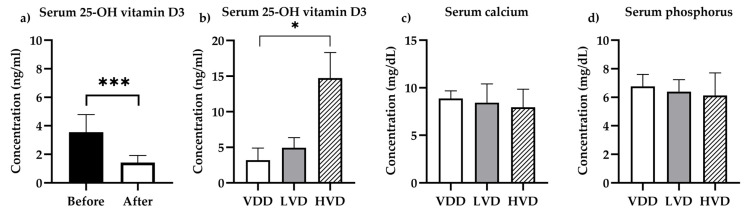
Vitamin D status and blood chemistry. (**a**) Serum levels of 25-OH vitamin D3 before (day 1) and after 4 weeks (day 28) of starting paricalcitol administration (n = 6); (**b**) serum levels of 25-OH vitamin D3 after 7 weeks of special diet administration; (**c**) serum calcium levels at the end of the experiment; (**d**) Serum phosphorus levels at the end of the experiment. Legend: VDD—vitamin D deficient group (n = 12), LVD—vitamin D deficient group supplemented with a special diet containing 1000 IU/kg cholecalciferol (n = 12), HVD—vitamin D deficient group supplemented with a special diet containing 10,000 IU/kg cholecalciferol (n = 12), *—*p* < 0.05, ***—*p* < 0.001.

The first step was to determine the concentration of 25-hydroxyvitamin D3 and cholecalciferol in the serum and brain homogenates before and after paricalcitol administration. Compared with the serum levels determined before treatment, rats administered with paricalcitol showed a significant decrease in serum 25-OH-D3 (Figure 4a, *p* < 0.001) and cholecalciferol levels. Moreover, the cholecalciferol levels in serum were below the detection level of the quantification method. 

After 7 weeks of vitamin D3 supplementation, a significant difference between the treatment groups was found regarding the serum and brain homogenate concentrations. Figure 4b illustrates that the 25-OH-D3 levels increased after a 7-week supplementation; however, a significant increase was observed in the HVD group only. Serum 25-OH-D3 increased to a mean (SEM) concentration of 14.72 (3.59) ng/mL compared with 3.201 (0.594) ng/mL in the VDD group, representing a more than 4-fold increase (Tamhane’s T2 multiple comparisons test, *p* = 0.028, Figure 4b). 

#### 3.1.2. Biochemistry

Serum calcium and phosphorus levels are crucial for cell homeostasis, including the CNS. It was therefore crucial to examine how paricalcitol-induced vitamin D deficiency and cholecalciferol supplementation impacted electrolytes and liver and kidney functions. Serum calcium and phosphorus levels showed no difference between the groups (Figure 4c,d, F (2, 20) = 0.6650, *p* = 0.525 and F (2, 20) = 0.6089, *p* = 0.554, respectively). Blood glucose, liver function, and kidney function were not affected (Table 1).

### 3.2. Behavioral Assays

#### 3.2.1. Open Field (OF) Test

**Figure 5:**
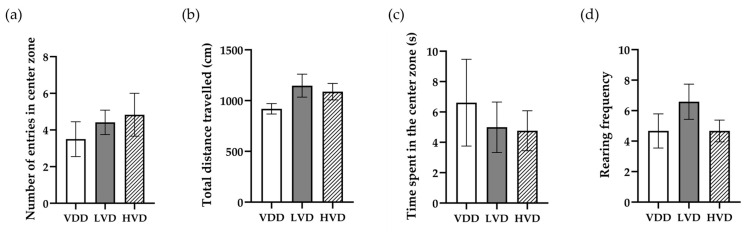
Assessment of locomotion and anxiety-like behavior in the open field test. (**a**) The number of entries in the center zone, defined as a 30 × 30 cm^2^ out of a 60 × 60 cm^2^ total surface of the arena; (**b**) the total distance travelled by the animal during the trial; (**c**) the total time spent in the center zone of the arena; (**d**) the vertical exploration of the animals was measured by counting the number of supported rearings during the 5-min-long trial. Data are expressed as mean ± SEM. Legend: VDD—vitamin D deficient group (n = 12), LVD—vitamin D deficient group supplemented with special diet containing 1000 IU/kg cholecalciferol (n = 12), HVD—vitamin D deficient group supplemented with special diet containing 10,000 IU/kg cholecalciferol (n = 12).

The OF test revealed no significant differences between the groups in terms of general locomotor ability and anxiety-like behavior. However, we noticed a slight increase in the number of entries in the center area as a result of cholecalciferol supplementation (VDD 3.5 ± 0.95 vs. LVD 4.42 ± 0.67 vs. HVD 4.83 ± 1.17, *p* = 0.41, Figure 5a), as well as in the total distance travelled during the trial (VDD 918.9 ± 51.18 vs. LVD 1147 ± 113.1 vs. HVD 1088 ± 81.1, *p* = 0.18, Figure 5b). The time spent in the center zone of the test area was not different between the groups (VDD 6.61 ± 2.86 vs. LVD 4.99 ± 1.66 vs. HVD 4.76 ± 1.32, *p* = 0.95, Figure 5c). Vertical exploration expressed as supported rearing also did not show statistically significant differences (VDD 4.67 ± 1.12 vs. LVD 6.58 ± 1.15 vs. HVD 4.67 ± 0.71, *p* = 0.25, Figure 5d). 

#### 3.2.2. Novel Object Recognition (NOR) Test

**Figure 6:**
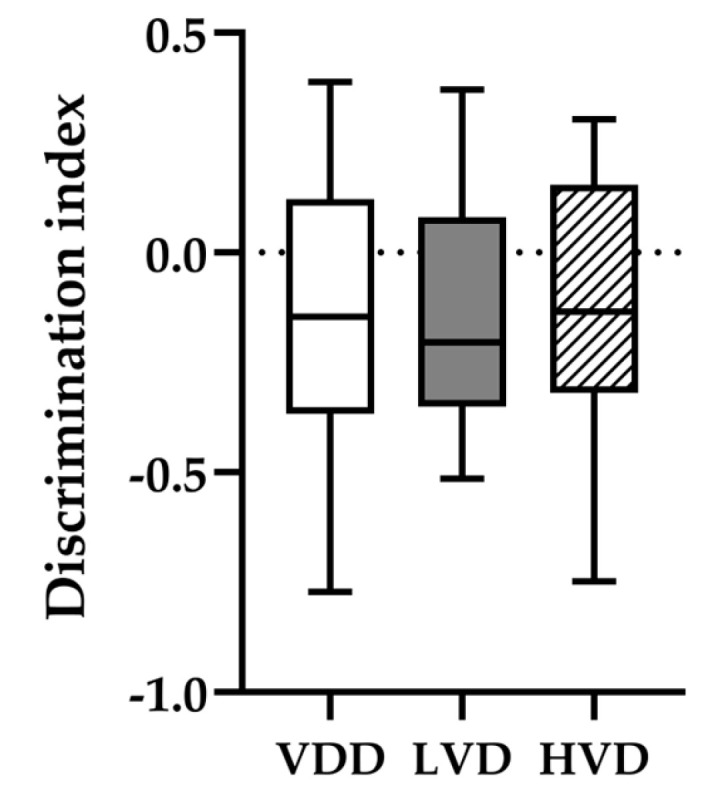
Discrimination index (DI) calculated for the retention trials of the NOR test. Line: median; box: interquartile range; whiskers: min to max. Legend: VDD—vitamin D deficient group (n = 12), LVD—vitamin D deficient group supplemented with a special diet containing 1000 IU/kg cholecalciferol (n = 12), HVD—vitamin D deficient group supplemented with a special diet containing 10,000 IU/kg cholecalciferol (n = 12).

According to the statistical analysis (ordinary one-way ANOVA), cholecalciferol supplementation did not have a significant impact on the discrimination index of novel object exploration (VDD −0.151 ± 0.1 vs. LVD −0.142 ± 0.08 vs. HVD −0.137 ± 0.09, *p* > 0.99, Figure 6). In addition, all three groups displayed a preference for exploring the familiar object rather than the novel one, as indicated by the negative discrimination indices. These findings indicate that at 4 h following pre-training, the rats that received vitamin D supplementation did not distinguish between the novel and the familiar object any better than the control animals.

#### 3.2.3. Morris Water Maze (MWM) Test

**Figure 7:**
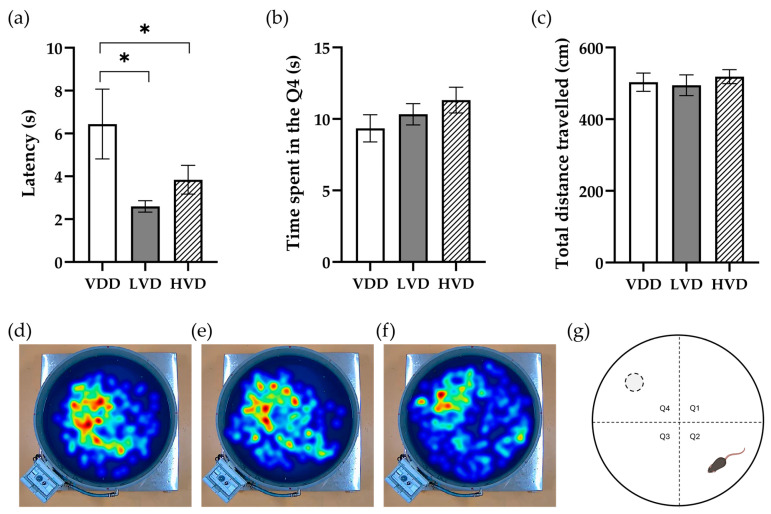
Assessment of spatial learning and memory in the testing phase of the Morris water maze. (**a**) Latency to reach the former platform location; (**b**) time spent in the target quadrant, which was the fourth quadrant of the tank; (**c**) total distance travelled during the 30-s retention swimming trial; (**d**–**f**) heatmap representation of swim paths of all three experimental groups. The heatmaps are based on the tracking data from Ethovision where the subject’s position is given by an *x*, *y* coordinate for each time point. Red color indicates that more time was spent in that area while blue color indicates less time; (**g**) schematic representation of the Morris water maze tank with the former platform location (grey circle surrounded by a dashed line) and the animal’s starting position (rat) marked. Data are expressed as mean ± SEM. Legend: VDD—vitamin D deficient group (n = 12), LVD—vitamin D deficient group supplemented with a special diet containing 1000 IU/kg cholecalciferol (n = 12), HVD—vitamin D deficient group supplemented with a special diet containing 10,000 IU/kg cholecalciferol (n = 12). Asterisk indicates the statistical significance level of * *p* < 0.05. The four quadrants in the water maze were denoted as Q1, Q2, Q3, and Q4.

The performance in the spatial learning task in the cholecalciferol-supplemented rats was significantly better than that of vitamin D-depleted control rats. This difference was indicated by the shorter latency it took the treated animals to reach the platform location (VDD 6.44 ± 1.63 vs. LVD 2.59 ± 0.57 vs. HVD 3.84 ± 0.67, *p* = 0.042, Figure 7a). We also measured the total time spent in the target quadrant. Although the difference in this parameter did not reach the significance level, we observed a trend of the treated animals towards spending more time in the target quadrant (VDD 9.34 ± 0.95 vs. LVD 10.33 ± 0.74 vs. HVD 11.32 ± 0.90, *p* = 0.28, Figure 7b). The total distance traveled did not vary between the three groups (VDD 503.3 ± 25.46 vs. LVD 494.7 ± 29.2 vs. HVD 518.8 ± 19.55, *p* = 0.79, Figure 7c).

#### 3.2.4. Radial Arm Maze Test (RAM)

**Figure 8:**
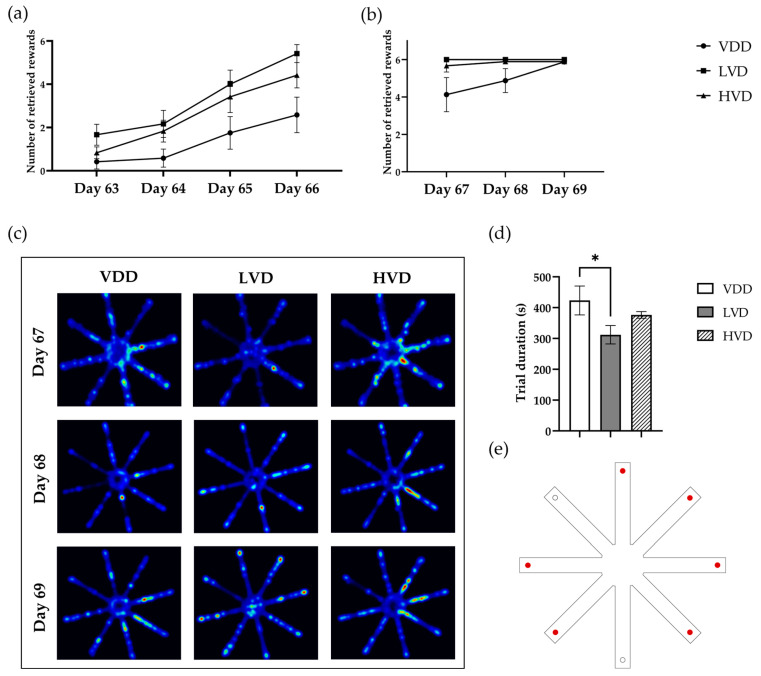
Results of the RAM test. (**a**) The number of collected food rewards during the training phase of the RAM test; (**b**) the number of collected food rewards during the testing phase of the RAM test; (**c**) heatmap visualization of the track plots of each experimental group across the three testing days. The heatmaps are based on the tracking data from Ethovision where the subject’s position is given by an *x*, *y* coordinate for each time point. Blue indicates that the rat spends less time in that area of the maze, while red demarcates where the rat spends the majority of its investigative time; (**d**) trial duration; (**e**) schematic representation of the radial arm maze with the sequence of baiting marked (red dot, baited arm; clear dot, unbaited arm). Data are expressed as mean ± SEM. Legend: VDD—vitamin D deficient group (n = 12), LVD—vitamin D deficient group supplemented with a special diet containing 1000 IU/kg cholecalciferol (n = 12), HVD—vitamin D deficient group supplemented with a special diet containing 10,000 IU/kg cholecalciferol (n = 12). Asterisk indicates the statistical significance level of * *p* < 0.05.

Over the course of four consecutive days of training in the radial arm maze, a between-group analysis revealed a significant effect of treatment on the total number of retrieved food rewards [F (2, 33) = 4.921, *p* = 0.0135, Figure 8a]. Furthermore, a significant within-group effect of time was observed [F (1.950, 64.34) = 38.19, *p* < 0.0001], indicating that all the animals eventually learned the task, with those receiving cholecalciferol supplementation exhibiting significantly faster learning rates. 

Regarding the performances during the testing period, rats in the VDD group performed significantly more poorly compared with the LVD and HVD groups [F (2, 24) = 5.33, *p* = 0.012, Figure 8b]. We found no differences between the LVD and HVD groups due to the high success rate of almost every animal in completing the task within the given timeframe. Consequently, we decided to measure and compare the total time required for the animals to complete the test over the 3-day testing period. A one-way repeated measures ANOVA analysis revealed that the LVD group was able to collect all the food rewards significantly faster than the VDD group (423.4 ± 46.8 vs. 312 ± 29.79, *p* = 0.027, Figure 8d). The HVD group was also slightly faster compared with the VDD group; however, this difference was not statistically significant (423.4 ± 46.8 vs. 376.5 ± 10.93, *p* = 0.28, Figure 8d). 

**Figure 9:**
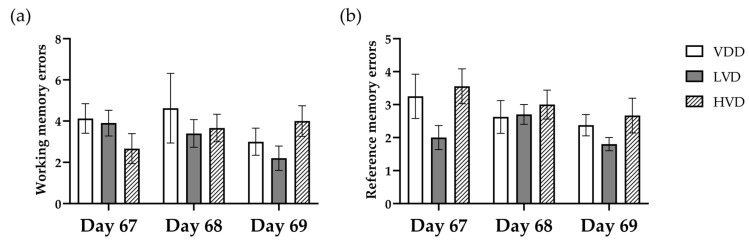
Number of working memory errors (**a**) and reference memory errors (**b**) made while looking for the food rewards across the 3 days of the RAM test. Data are expressed as mean ± SEM. Legend: VDD—vitamin D deficient group (n = 12), LVD—vitamin D deficient group supplemented with a special diet containing 1000 IU/kg cholecalciferol (n = 12), HVD—vitamin D deficient group supplemented with a special diet containing 10,000 IU/kg cholecalciferol (n = 12).

Working memory errors were assessed as the number of reentries into the arms already entered during the same trial. A two-way ANOVA analysis revealed that there was no significant between-group effect of treatment on the number of working memory errors [F (2, 24) = 0.402, *p* = 0.673, Figure 9a]. Similarly, we found no significant effect of cholecalciferol supplementation on the number of reference memory errors (2.75 ± 0.26 vs. 2.17 ± 0.27 vs. 3.07 ± 0.26, *p* = 0.25, Figure 9b), which were assessed as entries into an arm that never contained a reward.

### 3.3. Immunohistochemical Analysis

#### 3.3.1. Effects of Vitamin D3 Supplementation on the Cellular Reorganization of the Hippocampus

**Figure 10:**
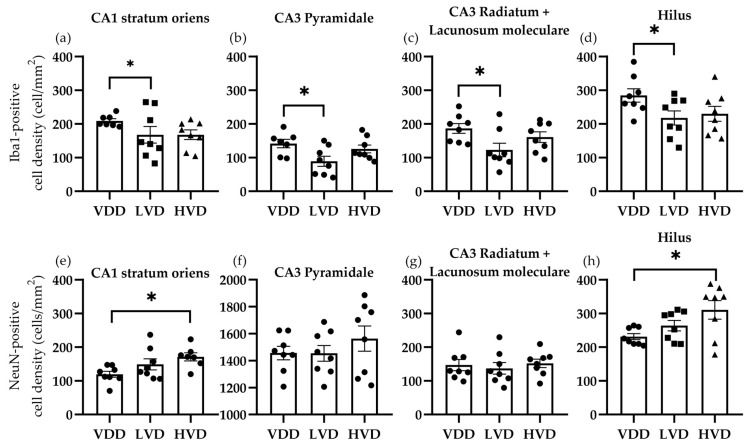
Microglial (Iba1-positive) and neuronal (NeuN-positive) cell density in the hippocampal subregions of vitamin D deficient and cholecalciferol-supplemented rats. In (**a**) the stratum oriens of the cornu Ammonis 1 subregion, (**b**) the stratum pyramidale, (**c**) the radiatum and lacunosum moleculare of the cornu Ammonis 3, and (**d**) in the hilus of the dentate gyrus, significant differences in microglia cell density were found. Conversely, neuron cell density displayed significant differences in (**e**) the stratum oriens of the cornu Ammonis 1 subregion and (**h**) in the hilus of the dentate gyrus, while (**f**) the stratum pyramidale, (**g**) the radiatum and lacunosum moleculare of the cornu Ammonis 3 did not differ. Data are expressed as mean ± SEM (n = 8). Legend: CA1—cornu Ammonis 1, CA3—cornu Ammonis 3, VDD—vitamin D deficient group, LVD—vitamin D deficient group supplemented with a special diet containing 1000 IU/kg cholecalciferol, HVD—vitamin D deficient group supplemented with a special diet containing 10,000 IU/kg cholecalciferol, *—*p* < 0.05.

**Figure 11:**
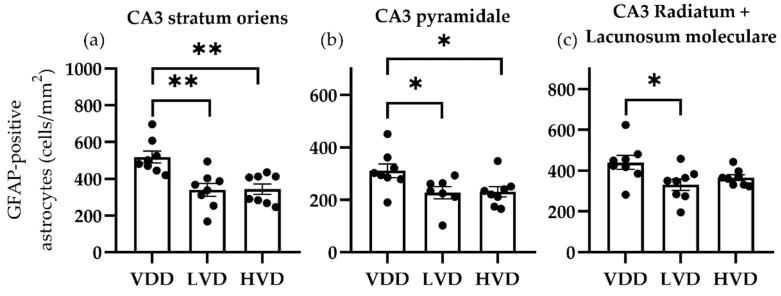
Astrocyte (GFAP-positive) cell density in the hippocampal subregions of vitamin D deficient and cholecalciferol-supplemented rats. All layers of the cornu Ammonis 3 subregion, (**a**) the stratum oriens, (**b**) the pyramidale, (**c**) the radiatum and lacunosum moleculare displayed significant differences between groups. Data are expressed as mean ± SEM (n = 8). Legend: CA3—cornu Ammonis 3, VDD—vitamin D deficient group, LVD—vitamin D deficient group supplemented with a special diet containing 1000 IU/kg cholecalciferol, HVD—vitamin D deficient group supplemented with a special diet containing 10,000 IU/kg cholecalciferol, *—*p* < 0.05, **—*p* < 0.01.

The following regions of interest were analyzed in the hippocampal area: all the layers of the CA1 and CA3 regions (i.e., stratum oriens, pyramidale, radiatum, and lacunosum moleculare) and the polymorphic layer of the dentate gyrus (i.e., hilus). The most important finding was that 1000 IU/kg of vitamin D3 supplementation significantly decreased the mean (SEM) Iba1-positive cell density in the CA1 stratum oriens (VDD vs. LVD vs. HVD, 208.9 ± 6.35 vs. 167.5 ± 24.64 vs. 167.8 ± 14.31, *p*= 0.0455, Figure 10a), the CA3 pyramidal layer (141.8 ± 12.60 vs. 88.94 ± 14.85 vs. 125.7 ± 11.74 cell/mm^2^, *p* = 0.0303, Figure 10b), the CA3 radiatum + lacunosum moleculare layers (186.4 ± 14.33 vs. 122.6 ± 19.85 vs. 160.8 ± 15.55 cell/mm^2^, *p* = 0.0427, Figure 10c), and the hilar region (284.4 ± 17.36 vs. 217.6 ± 19.53 vs. 229.8 ± 17.40 cell/mm^2^, *p* = 0.03, Figure 10d). In parallel, the NeuN-positive cell density was increased by vitamin D3 supplementation in the CA1 stratum oriens (119.4 ± 8.88 vs. 148.7 ± 16.35 vs. 171.1 ± 11.92 cell/mm^2^, *p* = 0.0347, Figure 10e) and the hilar region (231.0 ± 17.32 vs. 263.6 ± 22.10 vs. 310.3 ± 24.48 cell/mm^2^, *p* = 0.04, Figure 10h), while CA3 showed no differences between the treatment groups. Interestingly, GFAP-positive cells showed significant differences in the CA3 region, with both of the vitamin D3-supplemented groups showing a significant decrease in the astrocyte cell density (Figure 11). 

#### 3.3.2. Effects of Vitamin D3 Supplementation on the Cellular Reorganization of the Amygdala

**Figure 12:**
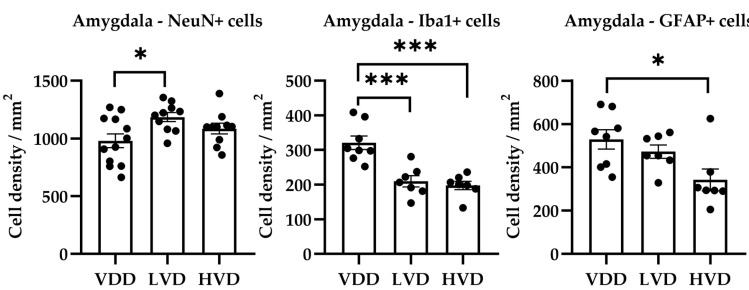
Neuronal (NeuN-positive), microglial (Iba1-positive), and astrocytic (GFAP-positive) cell density in the amygdala of vitamin D deficient and cholecalciferol-supplemented rats. Data are expressed as mean ± SEM (n = 8–12). Legend: VDD—vitamin D deficient group, LVD—vitamin D deficient group supplemented with a special diet containing 1000 IU/kg cholecalciferol, HVD—vitamin D deficient group supplemented with a special diet containing 10,000 IU/kg cholecalciferol, *—*p* < 0.05, ***—*p* < 0.001.

The VDD group showed a significantly higher mean (SEM) microglia cell density compared with the LVD and HVD groups (VDD 320.9 ± 19.55 vs. LVD 209.4 ± 16.23 vs. HVD 197.4 ± 12.26 cell/mm^2^, F (2, 19) = 17.34, *p* < 0.001) and a decreased neuron density compared with the LVD group (VDD 980.1 ± 60.13 vs. LVD 1185 ± 38.97 cell/mm^2^, F (2, 29) = 4.138, *p* = 0.03). The GFAP-positive cell density showed a significant difference between the VDD and HVD groups, with vitamin D3 supplementation decreasing the astrocyte density compared with the VDD group (Figure 12).

## 4. Discussion

The involvement of vitamin D signaling in the development and function of the brain has gained increasing attention recently. This study contributes to the understanding of how vitamin D3 supplementation might influence the cognitive performance of rats that exhibit VDD. The most important findings of this study are that vitamin D3 supplementation improved the learning ability of vitamin D-deficient adult rats in spatial task tests and induced cellular reorganization in the amygdala and hippocampus.

A variety of genetic knockout models and dietary deficiency models have been used to study the behavioral effects of manipulating vitamin D signaling on brain development and function [33,34,35]. Even though genetic models are more specific, dietary deficiency models have more construct validity. Rats housed in UV-free conditions and fed a vitamin D-deficient diet for 6 weeks will develop VDD [36]. This procedure consistently decreased the serum 25-OH vitamin D3 levels. In most of the studies, no differences in calcium and phosphate serum levels were reported if the calcium intake was increased [20,37]. However, vitamin D3 depletion can be accelerated by administering paricalcitol to rats in parallel with a vitamin D-deficient diet with a high calcium content [25]. Paricalcitol increases the expression of the 24-hydroxylase enzyme, which transforms both 1,25-dihydroxy vitamin D3 and 25-hydroxy vitamin D3 into their inactive forms [38]. Consequently, VDD induction can be shortened. This study supports the idea that paricalcitol can induce VDD in all rats without affecting calcium/phosphorus homeostasis or liver/kidney function.

Behavioral studies focusing on transient VDD and vitamin D3 supplementation in adults have been conducted using a variety of preclinical models. This study used the MWM, RAM, and NOR tests to assess the cognitive functions of rats and found that the HVD and LVD groups demonstrated a better performance in the MWM and RAM tests compared with the VDD group. Specifically, in the MWM test vitamin D3 supplementation reduced the latency to platform, the main parameter of this test. This is in accordance with previous research performed by Latimer et al., showing that high doses (10,000 IU/kg) of vitamin D3 decreased the latency to platform in the MWM task [23]. Regarding the RAM test, it can be noted that neither the working nor the reference memory were affected by vitamin D3 supplementation in the current work. However, the learning curve significantly differed between the groups, with the number of retrieved baits being higher and the trial duration being shorter in vitamin D3-supplemented groups compared with the VDD controls. Previous studies also showed that hippocampal lesions induced by lysophosphatidyl choline injection alter rats’ RAM test performance, but vitamin D3 supplementation can ameliorate this cognitive deficit [39]. Moreover, Tarbali and Khezri found that vitamin D3 supplementation improved rats’ working and reference memory, but their animal model exhibited a more profound cognitive deficit due to toxic demyelination, as reflected by a higher number of reference memory errors than in the current study [39]. In the NOR test, it can be noted that the discrimination index was negative in all the treatment groups, suggesting that the recognition memory was affected by VDD and vitamin D3 supplementation did not enhance this function. Although Alrefaie and Moustafa found that vitamin D3 supplementation improved recognition memory in high-fat fed Wistar rats, there are major differences in the study designs. They did not induce vitamin D deficiency and did not assess animals’ vitamin D status after supplementation [40]. In summary, the different types of cognitive functions (i.e., recognition memory, working memory, reference memory, learning, and visuospatial skills) assessed in this study were differently affected by vitamin D3 supplementation. Spatial learning, which relies on hippocampal function, was significantly improved. However, the novel object preference, which involves the prefrontal and perirhinal cortex, was not improved.

This study investigated the influence of vitamin D3 supplementation on the reorganization of neuron–astrocyte–microglia triad in VDD adult rats. The results showed that vitamin D3 supplementation caused a significant decrease in microglia cell density and increased the neuron cell density in the hippocampus and amygdala. The GFAP-positive astrocyte population also showed a significant decrease in the amygdala and in the CA3 region of the hippocampus. These findings suggest that vitamin D supplementation might ameliorate the progression of neuroinflammation and neuronal loss triggered by VDD. Cellular reorganization of the hippocampus and amygdala was described in many different neurological disorders, especially in neurodegenerative diseases such as Alzheimer’s and Parkinson’s disease, but it can also be found in epilepsy, stroke, and dementia [41,42,43,44]. Especially, the neuron–astrocyte–microglia triad shows alterations in aging associated cognitive decline and neuroinflammation [45,46,47]. The multiple roles of calcitriol in modulating apoptosis [48], neuroinflammation, oxidative stress [49], and microglia activation [50,51] were described earlier. VDD was proposed to accelerate aging and age-related diseases by contributing to the progression of neuronal loss and gliosis [52], whereas calcitriol exhibits neuroprotective actions [53] and decreases the levels of inflammation and degeneration markers [54,55]. The central role of the microglia in the initiation of neuroinflammation was elucidated previously. Its activation can occur due to several environmental (e.g., infection, trauma, and drugs) and endogenous factors (e.g., genetic mutation and protein aggregation), including VDD [56]. As they are the main innate immune cells of the CNS and the first responders to pathological insults, they produce proinflammatory cytokines, such as tumor necrosis factor (TNF)-α, interleukin (IL)-1β, IL16, and various chemokines to recruit additional immune cells in order to eliminate the pathological agent [57]. While neuroinflammation is primarily a neuroprotective mechanism, a sustained state of neuroinflammation can lead to neurotoxicity and neurodegeneration [58]. Vitamin D3 was shown to affect the activation of the microglia by modulating the expression of pro-inflammatory factors. Calvello et al. demonstrated that vitamin D treatment attenuates neuroinflammation via shifting pro-inflammatory (M1) microglia signaling to an alternative activation of the microglia for anti-inflammatory (M2) signaling [59]. It must be mentioned that according to today’s scientific understanding, the use of M1/M2 nomenclature is not entirely accurate. The changes in the phenotypes of the microglia, their loss of neuroprotective functions, and their gain of neurotoxic functions are complicated, so a simple classification does not capture the full range of phenotypes. They should be considered as being on a spectrum, instead of being viewed as two distinct populations [60]. Nevertheless, the results published by Calvello et al. provide evidence and support for the idea that vitamin D3 exerts immunoregulatory properties. Thus, the results of the current study support the hypothesis that the administration of vitamin D3 supplements might prevent cognitive decline and neurodegeneration in VDD subjects.

To date, there is no consensus regarding the therapeutic and supraphysiological doses of vitamin D3, and quite a wide range of doses have been tested in both preclinical and clinical assays. The cholecalciferol doses that we used in our research were selected based on previous investigations demonstrating that the administration of cholecalciferol up to 10,000 UI/kg diet in adult male rats (approximately 400 IU/day) is safe and free from adverse effects [22,23]. We observed no significant differences between the lower and higher doses of vitamin D3 supplementation in terms of the biochemical parameters, the performance in the neurocognitive tests, or the cellular reorganization of the hippocampus and amygdala. The fact that a ten-times-higher dose was unable to achieve significantly better outcomes raises intriguing questions regarding the therapeutic window of cholecalciferol and whether the damages caused by VDD can be reversed any better than this. On the other hand, according to the current understanding of vitamin D physiology in humans and rodents, a direct dose conversion is not possible.

To the best of our knowledge, the correlation between the cognitive enhancement and vitamin D metabolite concentrations that we have observed in a preclinical animal model of transient vitamin D deficiency has never been studied before. Therefore, our results provide new evidence supporting the association of cognitive impairment and insufficient or deficient levels of vitamin D, the modulatory effects on the microglia, and the neuroprotective effects of vitamin D supplementation. These findings have significant implications for human health, as they suggest that maintaining adequate levels of vitamin D may play a crucial role in preserving cognitive function and protecting against cognitive impairment. In spite of the existing evidence showing a positive correlation between serum 25-OH vitamin D3 and cognitive function in older adults, only a few randomized clinical trials have been conducted [61]. Because those studies are conflicting, further research on human subjects is needed to validate and explore the potential therapeutic interventions involving vitamin D supplementation.

The limitations of the current study include the use of only male rats and the lack of a morphological analysis of the microglia and astrocytes. The reason for choosing only males instead of females or mixed groups was because of the better spatial ability and reduced performance variation caused by hormonal fluctuations [62,63]. Due to technical limitations, only the cell density was analyzed. On the other hand, the microglia’s morphology cannot be considered a relevant feature to decide whether it plays a proinflammatory or anti-inflammatory role. 

## 5. Conclusions

Vitamin D3 supplementation improved the cognitive performance of vitamin D-deficient male rats in MWM and RAM tests associated with cellular changes in the amygdala and hippocampus. The decreased microglia and astrocyte cell density along with the increased neuron cell density in key regions of the hippocampus (i.e., hilus and CA3) suggest a protective role for vitamin D3 supplementation in VDD rats. Lower doses of vitamin D3 were sufficient to restore cognition and prevent cellular changes. Further studies are warranted in females to clarify the beneficial or maybe potentially harmful effects of vitamin D3 in rats. Additionally, the effects of vitamin D3 on brain inflammation and neuroprotection should also be investigated. Moreover, the potential clinical significance of these results should also be explored in human subjects.

## Figures and Tables

**Table 1:** Biochemical parameters determined from the serum samples at the end of the experiment.

Parameter	Treatment Group	Mean	Standard Deviation	One-Way ANOVA
Alanine aminotransferase ALT	VDD	24.13	4.190	F (2, 20) = 0.07007
	LVD	22.14	9.616	*p* = 0.9326
	HVD	23.88	15.83	
Aspartate aminotransferase AST	VDD	100.0	21.40	F (2, 20) = 1.025
	LVD	126.0	44.94	*p* = 0.3770
	HVD	109.9	36.96	
Alkaline phosphatase	VDD	122.9	38.52	F (2, 20) = 0.3936
	LVD	147.0	24.28	*p* = 0.6797
	HVD	136.3	77.34	
Creatinine	VDD	0.325	0.071	F (2, 20) = 2.103
	LVD	0.300	0.100	*p* = 0.1482
	HVD	0.238	0.092	
Total cholesterol	VDD	68.68	15.51	F (2, 20) = 0.7690
	LVD	58.36	11.07	*p* = 0.4767
	HVD	63.60	19.84	
Blood glucose	VDD	295.5	58.27	F (2, 18) = 0.8933
	LVD	253.6	53.81	*p* = 0.4267
	HVD	269.7	73.24	
Potassium	VDD	7.25	0.369	F (2, 17) = 0.1295
	LVD	7.39	0.501	*p* = 0.8794
	HVD	7.31	0.649	
Sodium	VDD	148.4	0.419	F (2, 24) = 1.915
	LVD	148.8	0.350	*p* = 0.1692
	HVD	148.8	0.748	

## Data Availability

The data presented in this study are available on request from the corresponding author. The data are not publicly available due to ethical and institutional restrictions on data sharing.
